# Aortoesophageal fistula and arch pseudoaneurysm after removing of a swallowed chicken bone: a case report of one-stage hybrid treatment

**DOI:** 10.1186/s12893-018-0335-1

**Published:** 2018-01-11

**Authors:** Jia-yu Shen, Hong-wei Zhang, Kang-jun Fan, Hu Liao, Er-yong Zhang, Jia Hu

**Affiliations:** 1 Department of Cardiovascular Surgery, West China Hospital of Sichuan University, Guo Xue Alley 37#, Chengdu, 610041 People’s Republic of China; 2 Department of Thoracic Surgery, West China Hospital of Sichuan University, Guo Xue Alley 37#, Chengdu, 610041 China

**Keywords:** Aortoesophageal fistula, Pseudoaneurysm, Thoracic aorta, Endovascular treatment, Stent-graft

## Abstract

**Background:**

Aortoesophageal fistula (AEF) and arch pseudoaneurysm are rare complications induced by a foreign body, and considerable controversy remains regarding the appropriate management strategies. We herein report a successful one-stage hybrid treatment in a patient with AEF and arch pseudoaneurysm.

**Case presentation:**

The patient, a 40-year-old man, presented to the emergency room because of intense retrosternal discomfort for 3 days and hematemesis for 3 h. The esophagoscopy and thoracic enhanced computed tomography (CT) showed two irregular mural ulcers in the esophagus and a large saccular pseudoaneurysm at the aortic isthmus, respectively. The laboratory examinations confirmed no widespread inflammation and infection. We have successfully performed a successful one-stage hybrid treatment for this patient. Six-month follow-up shows the patient is in good condition and the esophagoscopy reveals the two mural ulcers had completely healed.

**Conclusion:**

The treatment decision-making process should depend upon the patients’ specific situations. Our case suggest the one-stage hybrid treatment could be an valuable alternative in some selected patients.

**Electronic supplementary material:**

The online version of this article (10.1186/s12893-018-0335-1) contains supplementary material, which is available to authorized users.

## Background

Accidental swallow of foreign bodies is a relatively common condition encountered in the emergency room, but it rarely causes severe complications. Although some may become entrapped within the esophagus, it can be safely and effectively managed with endoscopy [1]. Esophageal perforation and formation of a pathologic communication between the aorta and esophagus in the setting of a previous esophagoscopic treatment of impacted foreign body is particularly unusual. Here we present our successful experience in managing a patient with AEF and arch pseudoaneurysm late after removing of a chocked chicken bone by one-stage, multidisciplinary strategy of combined endovascular and open surgical approach.

## Case presentation

**Fig. 1:**
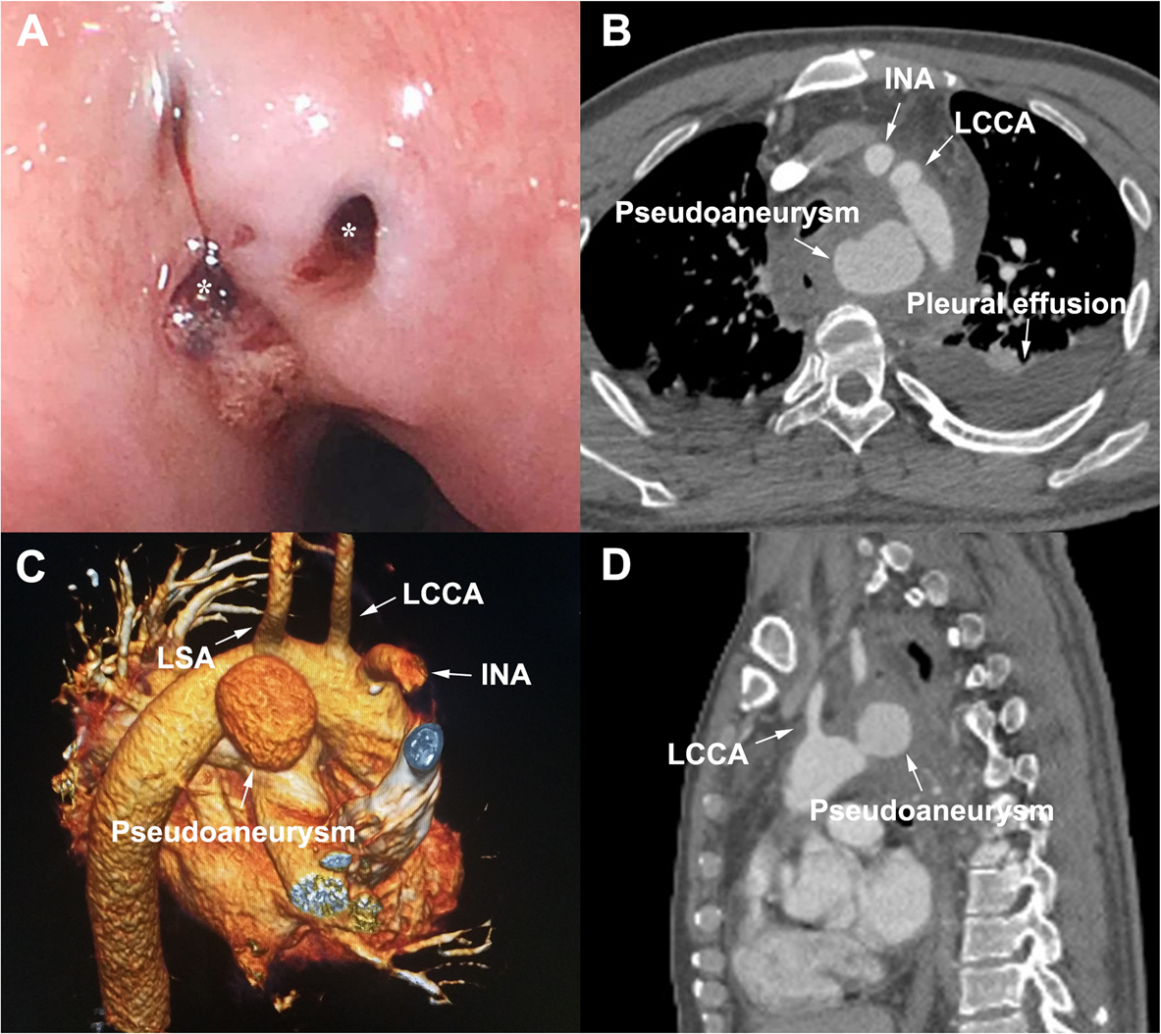
The pre-surgical examination: (**a**) Gastroscopic examination showed two esophageal fistulas approximately 0.2 cm and 0.3 cm in diameter respectively were found 23 cm away from the incisors; (**b**) Transverse, (**c**) three-dimensional volume-rendered and (**d**) sagittal computed tomography angiography demonstrated the pseudoaneurysm with a primary entry tear at the arch accompanied by gas-containing pleural effusion. *, irregular esophageal mural ulcers; LSA, left subclavian artery; LCCA, left common carotid artery; INA, innominate artery

A 40-year-old man presented to the emergency room because of intense retrosternal discomfort for 3 days and hematemesis for 3 h. The patient had a history of chocking chicken bone 1 month ago and was successfully managed by esophagoscopy at local district hospital. On admission, physical examination revealed body temperature of 37.1 °C, heart rate 111 beats/min, respiratory rate 20 per minute, and blood pressure 85/50 mmHg. Results of laboratory examinations demonstrated moderate anemia (hemoglobin 9.8 g/dl) and mild leukocytosis (leukocyte count 10.9 × 10^9^/L). The patient underwent esophagoscopy, which showed fresh and clotted blood coming from two irregular mural ulcers in the upper and middle thirds of the esophagus (Fig. 1a). Thoracic enhanced CT confirmed the presence of an AEF as well as a large saccular pseudoaneurysm at the aortic isthmus, accompanied by mediastinal hematoma and bilateral pleural effusion (Fig. 1b-d).

After multidisciplinary discussion, one-stage hybrid approach was accepted as reasonable and life-saving. Firstly, the patient underwent urgent aortic endovascular repair. A 12 mm double-disk vascular occluder (SHSMA Co. Ltd., Shanghai, China) was advanced into the long sheath then the pseudoaneurysm through its neck. Under fluoroscopic guidance, the anterior disc of the occluder was deployed in the pseudoaneurysm and pulled back gently against the rim of the neck. Further withdrawal of the sheath was made to release the waist of the occluder cross the neck and then the posterior disc in the aortic lumen. A thoracic stent graft (straight 30 mm × 20 cm; Valiant Captiva, Medtronic) was then introduced and deployed at the distal aortic arch covering the occluder and the ostium of the left subclavian artery. Angiography on completion revealed no leakage into the aneurysmal sac. An additional movie file shows this in more detail [see Additional file 1]. Subsequently, an exploratory left thoracotomy through the 5th intercostal space was performed. The pseudoaneurysm and adjacent hematoma was found below the azygos arch, and no signs of significant infection and active bleeding were found in the mediastinum. Intraoperative cultures were negative. After careful debridement, the dead space between the aorta and esophagus was filled with a viable pedicle flap of the omentum. A jejunostomy fistula tube and nasogastric tube were inserted for nutritional support and to drain digestive juice separately for 10 days. Two chest tubes were inserted for mediastinal irrigation and drainage.

**Fig. 2:**
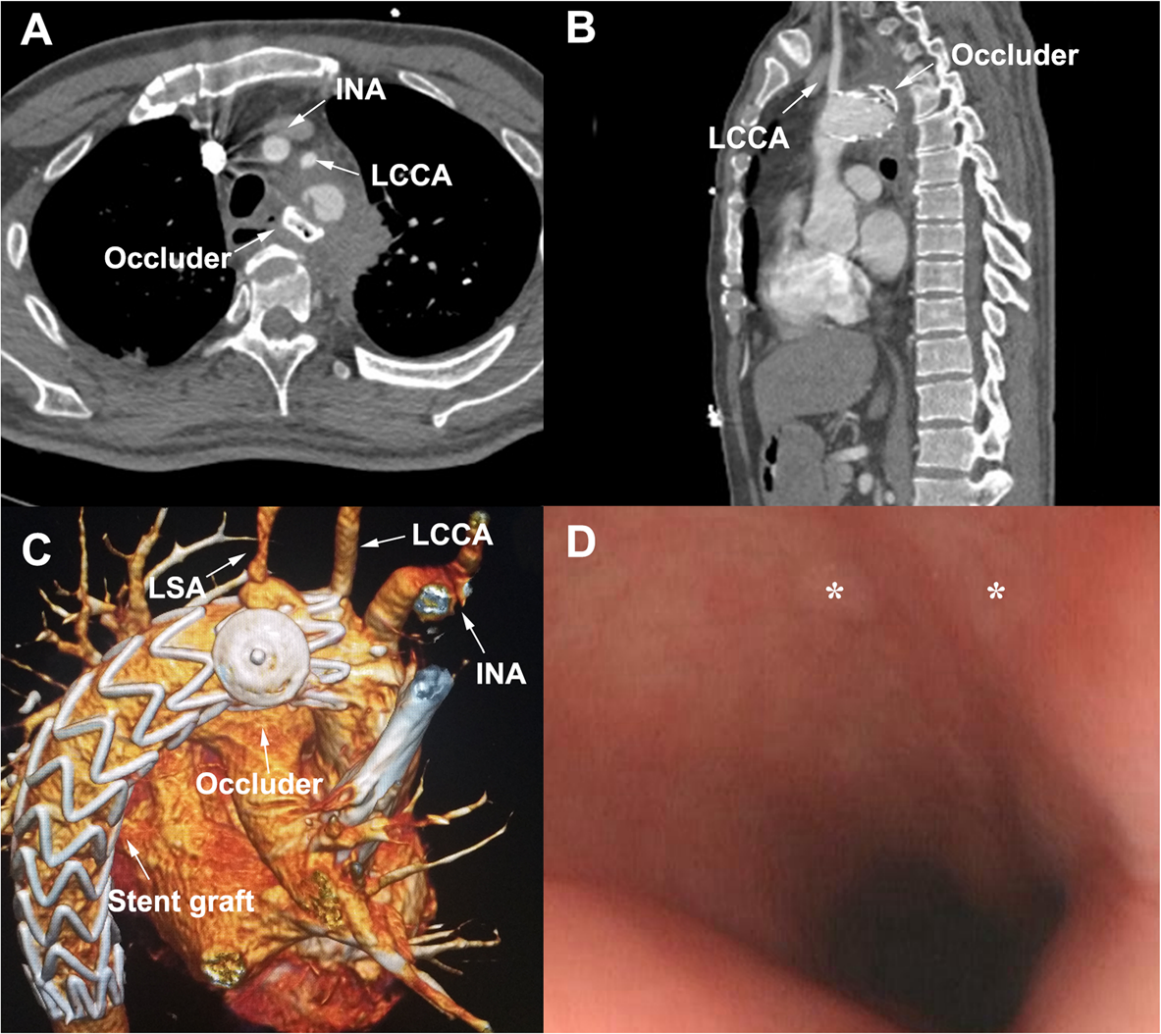
The post-surgical examination: (**a**) Transverse, (**b**) sagittal and (**c**) three-dimensional volume-rendered computed tomography angiography of pre-discharge showed complete sealing of the primary entry tear with the occlude. No endoleak could be found. **d** Gastroscopic examination revealed the complete healing of original fistulas. *, original esophageal mural ulcers; LCCA, left common carotid artery; LSA, left subclavian artery; INA, innominate artery

After surgery, the patient received fasting and antibiotic treatment (intravenous Imipenem and Cilastatn Sodium [0.5 g] once per 8 h for 4 weeks) and the intraoperative cultures were negative. Six months later, the postoperative CT scan showed well-positioned aortic prosthesis and no contrast extravasation from the aortic arch into the mediastinum and esophagus (Fig. 2a-c). The patient remains well and follow-up esophagoscopy showed that the two mural ulcers were completely healed (Fig. 2d).

## Discussion and conclusions

Esophageal perforation due to foreign body ingestion is relatively unusual in adults, and is rare when it causes an abnormal communication from the esophagus to aorta (0.08%–0.14%) [2–4]. Typical patients suffering from this complication were presented with a clinical triad of mid-thoracic chest pain, sentinel arterial hemorrhage and fatal hemorrhage, which was firstly summarized by Chiari [5] as “aorta-esophageal syndrome” or was also known as AEF.

The exact pathogenesis of AEF remains to be fully understood. Several studies suggested that esophageal ischemia secondary to elevated pressure in the posterior mediastinum, inflammation of the resorbed hematoma or mechanical compression by surrounding organs could be a causative factor [2, 6, 7]. In this case, impacted chicken bone could firstly lead to inflammation and ulceration of the esophageal wall. Moreover, the integrity of the adjacent aortic wall might also be impaired, which predisposed the formation of pseudoaneurysm. Although the chicken bone was removed uneventfully, pressure necrosis of the lacerated esophageal wall due to ongoing compression of the enlarged pseudoaneurysm would ultimately create an AEF late after initial impaction.

AEF is a devastating and usually life-threatening condition with a fatality rate of 40%–60% [2–4, 6, 7]. Conservative medical management of AEF patients almost invariably results in a fatal outcome [7]. Thus, surgical intervention was regarded as the only effective solution [1]. Traditional open repair includes combined replacement or bypass the involved aortic area of the fistula with concomitant repair or resection of the affected esophagus. However, the operative mortality was reported as high as 45.4–55% [8–10], which are due to the emergent nature of surgery, high risk of great vessel injury in an infected area, the need for aortic cross-clamping and significant blood loss.

Thoracic endovascular aortic repair (TEVAR) is less invasive than open surgery and enables rapid exclusion of aortic fistula and control of fatal hemorrhage. For AEF patients with unstable hemodynamics, TEVAR should be considered as a first-choice life-saving procedure [3, 4]. As our patient was admitted with hemorrhagic shock, we performed TEVAR at first without hesitation. However, the proximal landing zone was inadequate because of the short distance (<1.0 cm) between the neck of pseudoaneurysm and the ostium of left carotid artery. An occluder combined with aortic stent was used to facilitate a complete exclusion of the aneurysm sac. An innominate artery-to-left carotid artery-to-left subclavian artery bypass prior to the TEVAR procedure could be an alternative to obtain an adequate proximal landing zone. However, in this case, the instable hemodynamics prevented us from performing these time-consuming procedures. Moreover, the early trans-membrane leakage after stent deployment was another concern, and therefore we chose the occluder to secure a complete exclusion of the aneurysm.

Even if associated with favorable perioperative outcomes, TEVAR alone leaves the source of contamination (esophageal or bronchial perforation) untreated and incurs a risk of stent graft infection and/or fistula recurrence [1], which was also reported in the scenario of aortobronchial fistula [11]. Debate has begun on whether TEVAR can be used as a definitive procedure or as a bridging measure for further open esophagectomy and aortic replacement [4, 7]. In this case, an exploratory thoracotomy was performed immediately after TEVAR. Because no sign of infection in the mediastinum was detected, the esophageal fistula was not treated. Even though, omentopexy, which was reported to be highly useful to enhance healing process in an infected or fragile area [3, 7], was performed to occupy the dead space after hematoma debridement.

Based on our limited experience learned from this unique case, we suggested that management options for AEF patients should depend upon the location and extent of aortic involvement, severity of mediastinal infection and patients’ overall health status. For patients with initial hemodynamic stability, aortic intervention preferably with TEVAR should be done as soon as possible, because this condition can deteriorate rapidly once started bleeding. For patients with initial hemodynamic instability, various endovascular techniques could be used to restore the integrity of the aorta. Subsequently, an exploratory open surgery is important for further treatment decision-making. If patient do not exhibit widespread inflammation and infection, extensive debridement followed by omentopexy and abundant mediastinal irrigation seem to offer satisfactory results. Otherwise, simultaneous or staged esophagectomy and aortic replacement might be reliable for better outcomes of this condition. Adequate antibiotic treatment and lifelong imaging surveillance is mandatory, and a PET-imaging is a useful technique for these patients with any suspected signs of post-surgery infection.

AEF and arch pseudoaneurysm induced by a foreign body are rare but severe complications. The treatment decision-making process should depend upon the patients’ specific situation. Our practice indicates that the one-stage hybrid treatment could be a valuable alternative in some selected patients.

## Additional file

Additional file 1: The procedure of the placement of an occluder combined with aortic stent for pseudoaneurysm. (MP4 82,688 kb)

## Abbreviations

AEF: Aortoesophageal fistula
CT: Computed tomography
TEVAR: Thoracic endovascular aortic repair
